# Role of Two Cell Wall Amidases in Septal Junction and Nanopore Formation in the Multicellular Cyanobacterium *Anabaena* sp. PCC 7120

**DOI:** 10.3389/fcimb.2017.00386

**Published:** 2017-09-05

**Authors:** Jan Bornikoel, Alejandro Carrión, Qing Fan, Enrique Flores, Karl Forchhammer, Vicente Mariscal, Conrad W. Mullineaux, Rebeca Perez, Nadine Silber, C. Peter Wolk, Iris Maldener

**Affiliations:** ^1^Interfaculty Institute of Microbiology and Infection Medicine Tübingen, Organismic Interactions, University of Tübingen Tübingen, Germany; ^2^Instituto de Bioquímica Vegetal y Fotosíntesis, Consejo Superior de Investigaciones Científicas and Universidad de Sevilla Seville, Spain; ^3^Department of Microbiology-Immunology, Feinberg School of Medicine of Northwestern University Chicago, IL, United States; ^4^School of Biological and Chemical Sciences, Queen Mary University of London London, United Kingdom; ^5^MSU-DOE Plant Research Laboratory and Department of Plant Biology, Michigan State University East Lansing, MI, United States

**Keywords:** cyanobacteria, heterocysts, peptidoglycan, amidase, AmiC, septal junctions, cell–cell communication, SepJ

## Abstract

Filamentous cyanobacteria have developed a strategy to perform incompatible processes in one filament by differentiating specialized cell types, N_2_-fixing heterocysts and CO_2_-fixing, photosynthetic, vegetative cells. These bacteria can be considered true multicellular organisms with cells exchanging metabolites and signaling molecules via septal junctions, involving the SepJ and FraCD proteins. Previously, it was shown that the cell wall lytic *N*-acetylmuramyl-L-alanine amidase, AmiC2, is essential for cell–cell communication in *Nostoc punctiforme*. This enzyme perforates the septal peptidoglycan creating an array of nanopores, which may be the framework for septal junction complexes. In *Anabaena* sp. PCC 7120, two homologs of AmiC2, encoded by *amiC1* and *amiC2*, were identified and investigated in two different studies. Here, we compare the function of both AmiC proteins by characterizing different *Anabaena amiC* mutants, which was not possible in *N. punctiforme*, because there the *amiC1* gene could not be inactivated. This study shows the different impact of each protein on nanopore array formation, the process of cell–cell communication, septal protein localization, and heterocyst differentiation. Inactivation of either amidase resulted in significant reduction in nanopore count and in the rate of fluorescent tracer exchange between neighboring cells measured by FRAP analysis. In an *amiC1 amiC2* double mutant, filament morphology was affected and heterocyst differentiation was abolished. Furthermore, the inactivation of *amiC1* influenced SepJ localization and prevented the filament-fragmentation phenotype that is characteristic of *sepJ* or *fraC fraD* mutants. Our findings suggest that both amidases are to some extent redundant in their function, and describe a functional relationship of AmiC1 and septal proteins SepJ and FraCD.

## Introduction

The filamentous cyanobacterium *Anabaena* (*Nostoc*) sp. strain PCC 7120 (hereafter *Anabaena*) is a model organism to study and analyze multicellularity in prokaryotes. This photosynthetic, gram-negative bacterium grows in the form of filaments, composed of several 100 interacting cells, which exchange metabolites and signaling molecules with each other (Flores et al., 2006; Flores and Herrero, 2010). Under nitrogen limiting conditions, oxygen-producing vegetative cells differentiate into nitrogen-fixing heterocysts in a semi-regular pattern, a process controlled at the transcriptional level by the DNA-binding proteins NtcA and HetR (Muro-Pastor and Hess, 2012). Heterocysts undergo morphological and physiological changes to provide a micro-oxic environment for the oxygen-sensitive nitrogen fixation machinery (Maldener et al., 2014). Heterocysts supply the vegetative cells with metabolites derived from nitrogen fixation and, conversely, receive reduced carbon compounds derived from photosynthetic CO_2_-fixation (Wolk, 1968; Wolk et al., 1994; Kumar et al., 2010). Furthermore, heterocyst pattern formation along the filament requires exchange of signaling molecules (Yoon and Golden, 1998). Hence, growth and differentiation of a heterocyst-containing filament depend strictly on the exchange of regulatory compounds and metabolites.

An efficient cell–cell communication system along the filament is needed to enable rapid exchange of metabolites and regulators. In *Anabaena* two routes of intercellular molecular exchange have been suggested: one is via the continuous periplasm (Flores et al., 2006; Mariscal et al., 2007), the other by diffusion via cell–cell joining structures from cytoplasm to cytoplasm (Mullineaux et al., 2008). Poorly resolved cell-joining structures have long been known in the intercellular septa of heterocyst-forming cyanobacteria and had received different designations: microplasmodesmata, septosomes, septal junctions, or channels (Metzner, 1955; Wildon and Mercer, 1963; Giddings and Staehelin, 1981; Wilk et al., 2011; Mariscal, 2014; Omairi-Nasser et al., 2014; Flores et al., 2016). We will refer to cell–cell connections as septal junctions. Three membrane proteins, SepJ (FraG), FraC, and FraD, have been described that localize to the intercellular septa and are required for the establishment of long filaments in *Anabaena*. Mutants lacking *sepJ, fraC* or *fraD* show filament fragmentation and impaired intercellular molecular exchange measured by FRAP (fluorescence recovery after photobleaching) analysis (Flores et al., 2007; Nayar et al., 2007; Mullineaux et al., 2008; Merino-Puerto et al., 2010, 2011). It has been suggested that the SepJ and Fra proteins form septal junction complexes that traverse the septal peptidoglycan, thereby creating a route for intercellular communication (Nürnberg et al., 2015). These are integral membrane proteins that bear a predicted periplasmic domain (reviewed in: Herrero et al., 2016).

Circular perforations (termed nanopores) have been discovered in the septal walls of isolated peptidoglycan sacculi. About 100 to 150 of these nanopores are arranged in a semi-regular array in the central part of the septal peptidoglycan disks of *Nostoc punctiforme* (Lehner et al., 2013) and *Anabaena* (Nürnberg et al., 2015). These nanopores may be the sites where septal junctions traverse the septal peptidoglycan. In *N. punctiforme*, the *N*-acetylmuramyl-L-alanine amidase AmiC2 (ORF: *Npun_F1846*) is required for the formation of the septal peptidoglycan nanopores. Inactivation of the encoding gene, *amiC2*, results in severe phenotypic alterations with cells that show irregular cell-division planes, impaired cell differentiation and abolished intercellular molecular exchange, suggesting that formation of a nanopore array in the septal murein by AmiC2 is essential for intercellular communication (Lehner et al., 2011, 2013; Büttner et al., 2016). AmiC2 localizes to the newly formed septum and migrates during septal invagination to cover the entire septal plane. In older septa, however, the protein can no longer be detected. As in other heterocyst-forming cyanobacteria, the *amiC2* gene of *N. punctiforme* is located downstream of a very similar gene, *amiC1 (Npun_F1845)*, which could not yet be inactivated (Lehner et al., 2011; Faulhaber, 2017). In contrast, both amidases of *Anabaena* have been successfully inactivated. A mutant of *amiC1* (*alr0092*) was unable to form heterocysts and lost intercellular communication (Berendt et al., 2012). For *amiC2* (*alr0093*), two independent studies reported different results. Whereas, Berendt et al. (2012) found that the inactivation of *amiC2* showed no observable phenotypic alteration, Zhu et al. (2001) found that a mutant lacking *amiC2* (termed *hcwA*) could not grow diazotrophically (Fox^−^ phenotype). This seeming conflict was somewhat clarified when it was found that the DR1992 mutant of *amiC2* (*alr0093*), which bears a transposon within that gene, is Fox^−^ in AA/8 medium (Zhu et al., 2001) or BG110 medium, but the SR478 mutant of *amiC2*, which bears a single-recombination insertion in that gene, is Fox^+^ when grown in BG11_0_ but Fox^−^ incubated in AA/8 (our unpublished results). In GFP fusion-based localization studies in *Anabaena* and *N. punctiforme* both amidases, AmiC1 and AmiC2, localized to the septa of young vegetative cells and to the septal and polar neck areas connecting heterocysts with vegetative cells (Berendt et al., 2012; Faulhaber, 2017). Here, we present new insights into the function of both amidases of *Anabaena* and clarify the phenotypes of *amiC1* and *amiC2* mutants by multiple mutational approaches.

## Materials and methods

### Bacterial strains and growth conditions

*Anabaena* and its derivatives (Table S1) were grown either in BG11 medium containing NaNO_3_ or BG11_0_ medium lacking combined nitrogen (Rippka et al., 1979). Cultures were grown at 28 or 30°C in the light (30–40 μE m^−2^ s^−1^) in 100 ml Erlenmeyer flasks with constant shaking (100 r.p.m.) or on medium solidified with 1.5% (w/v) Difco Agar. Mutant strains were grown in media supplemented with antibiotics at the following concentrations: streptomycin (Sm), 5 μg ml^−1^; spectinomycin (Sp), 5 μg ml^−1^ and (or) neomycin (Nm), 50 μg ml^−1^. To induce heterocyst formation (nitrogen step-down), exponentially growing cultures were washed three times with BG11_0_ medium, resuspended in BG11_0_ medium equal to the original volume, and incubated under growth conditions. To keep the selective pressure on non-segregated mutant strains high and constant, cultures were weekly transferred to fresh medium containing the appropriate antibiotics. Chlorophyll *a* (Chl) content of cultures was determined according to Mackinney (1941).

*Escherichia coli* strains were grown in LB medium. When appropriate, the medium was supplemented with antibiotics at concentrations of 50 μg ml^−1^ kanamycin (Km), 25 μg ml^−1^ chloramphenicol (Cm), 25 μg ml^−1^ Sm and (or) 100 μg ml^−1^ Sp. Strain HB101 was used for conjugation with *Anabaena* (Wolk et al., 1984).

Mutant strains SR477 and SR478 (Berendt et al., 2012), DR1992 (Zhu et al., 2001), CSVT22 and CSVM34 (Merino-Puerto et al., 2011), and CSVM141 (Nürnberg et al., 2015) have been described previously. All strains are summarized in Table S1.

### Construction of *Anabaena* mutant strains

Plasmids (Table S1) were introduced into *Anabaena* strains by conjugation (Elhai and Wolk, 1988a). The *amiC1, amiC2, sepJ, fraC*, and *fraD* genes are open reading frames *alr0092, alr0093, alr2338, alr2392*, and *alr2393* of the *Anabaena* genome, respectively (Kaneko et al., 2001).

To delete an internal portion of *amiC1*, left- and right-flanking regions of *amiC1* were amplified with primers F7 and R11, and F11 and R7 (Table S2), respectively, using genomic DNA of *Anabaena* as template. PCR products were fused using primers F7 and R7. The product of amplification was cloned in pSpark (Canvax), yielding pCSAC10. Similarly, an internal portion of *amiC2* was deleted by amplifying left- and right-flanking regions of *amiC2* with primers F9 and R12, and F12 and R9, respectively (Table S2), using genomic DNA of *Anabaena* as template; the PCR products were fused using primers F9 and R9; and the product of amplification was cloned in pSpark, yielding pCSAC1. SacI-fragments from pCSAC1 and pCSAC10 were cloned in pCSRO (Corrales-Guerrero et al., 2013), producing pCSAC12 and pCSAC3, respectively. Plasmids pCSAC12 and pCSAC3 were used for conjugation as previously described. The *amiC1* mutant FQ1633 was generated by mutagenesis of *Anabaena* with transposon Tn*5*-1063, as described by Wolk et al. (1991). The *amiC1 amiC2* double mutant FQ1633 SR478 was generated by transferring plasmid pIM478 (Berendt et al., 2012) to mutant strain FQ1633. The *amiC1 amiC2* double mutant SR477 DR1992 was generated by transferring plasmid pIM477 (Berendt et al., 2012) to mutant strain DR1992 (Zhu et al., 2001). To test the activity of the *hetR* promoter, the self-replicating plasmid pCSAM195 (Alicia Muro-Pastor, unpublished) was transferred via conjugation to the *amiC1* mutant SR477 and to the wild-type strain. Plasmid pCSAM195 encodes the *hetR* promoter transcriptionally fused to a promoterless *e-gfp* gene allowing analysis of *hetR* promoter activity by fluorescence microscopy. To study the effect of inactivation of *amiC1* on the subcellular localization of SepJ-GFP, plasmid pCSVT22 bearing a *sepJ*-*gfp* fusion (Merino-Puerto et al., 2010) was transferred to the wild-type strain and strains SR477, CSAC1, and CSAC2 by conjugation. Because pCSVT22 is based on pRL424 (Elhai and Wolk, 1988b) that cannot replicate in *Anabaena*, it recombines with the chromosomal *sepJ* gene producing the functional *sepJ-gfp* fusion.

### Nitrogenase activity

Nitrogenase activity was determined by the acetylene reduction technique as described by Montesinos et al. (1995). In short, filaments grown in BG11 medium were harvested, washed three times with BG11_0_ medium, and resuspended in BG11_0_ medium. After 48 h of incubation under growth conditions, cell suspensions (5–20 μg Chl) were placed in flasks sealed with rubber stoppers and incubated under an atmosphere of 13.3% acetylene in air (oxic conditions). After 3–4 h of incubation with shaking in the light at 28°C (Tübingen) or 30°C (Seville), the amount of ethylene produced was determined by gas chromatography.

### Light and fluorescence microscopy

Light microscopy was performed with a Leica DM2500 B microscope connected, for color micrographs, with a Leica DFC420C camera. Images of fluorescence were taken with a Leica DM5500 B microscope with a 100x/1.3 oil objective lens (Leica Microsystems) connected with a Leica DFC360FX black and white camera. For visualization, specific filter cubes for fluorescence microscopy were needed depending on fluorophore spectral properties. GFP was monitored with a BP470 40-nm excitation filter and a BP525 50-nm emission filter. Cyanobacterial auto-fluorescence was monitored using a BP535 50-nm excitation filter and a BP610 75-nm emission filter. Bright-field images were exposed for 10 ms and 80 to 150 ms in the fluorescence channels. Images of fluorescence were re-colored by the Leica ASF software based on the filters used. Filament fragmentation was analyzed as previously described (Merino-Puerto et al., 2010). To stain the polysaccharide layer of heterocysts, cell suspensions were mixed with a 1% (w/v) solution of Alcian Blue (McKinney, 1953).

### Visualization of nanopores by transmission electron microscopy

Peptidoglycan (murein) sacculi were isolated from filaments grown in BG11 medium and analyzed as previously described (Lehner et al., 2013), with modifications. To get a higher yield of septal peptidoglycan, filaments were treated with a Branson Sonifier 250 (duty cycle: 50%; output control: 3) for 60 s to break up the filaments and to break off the lateral cell wall, which seems to be less stable than the septal peptidoglycan with its thick outer border. The purified sacculi were deposited on formvar/carbon film-coated copper grids (Science Services GmbH München), and stained with 1% (w/v) uranyl acetate. All of the samples were examined with a Philips Tecnai10 electron microscope at 80 kV.

### FRAP analysis

Staining of *Anabaena* with calcein or 5-carboxyfluorescein (5-CF) and FRAP measurements were performed as previously described (Mullineaux et al., 2008; Merino-Puerto et al., 2011). Filaments were harvested, washed three times with fresh medium, resuspended in 500 μl of fresh medium, mixed with 10 μl of either calcein acetoxymethylester (AM; 1 mg/ml in DMSO) or 5-CF diacetate AM (1 mg/ml in DMSO) and incubated in the dark for 90 min at 28°C. The samples were then washed three times with dye-free medium followed by a further dark incubation for 30 min. Filament suspensions were spotted onto BG11 agar and then covered with a cover slip. All measurements were carried out at room temperature with a Zeiss LSM 800 confocal microscope using a 63x/1.4 oil-immersion objective and the 488 nm line of a 10 mW laser as the excitation source. FRAP measurements were performed using the software ZEN 2.3 (blue edition). A 191-μm confocal pinhole (corresponding to 4.49 airy units) was used for imaging, giving a point-spread in the Z-direction of about 3 μm. Chl auto-fluorescence (emission detection: 650–700 nm) and either calcein or 5-CF fluorescence (emission detection: 400–530 nm) were imaged simultaneously. Imaging was done using the following settings: laser intensity: 0.2% (ensures no bleaching during image acquisition); frame size: 36.2 × 36.2 μm; pixel size: 0.07 μm; pixel dwell time: 1.52 μs; averaging: 1x line average. After capturing a sequence of initial pre bleach images, the fluorescence of a region of interest was bleached by a “fast-bleach” increasing laser intensity by a factor of at least 10 and recovery of the fluorescence signal was then recorded as a sequence of images at 1 s intervals for 30–60 s. FRAP data were analyzed using ImageJ version 1.49b (Schneider et al., 2012) for measuring the fluorescence intensity of a FRAP sequence, and data were processed with GraphPad PRISM version 6.01 for Windows (GraphPad Software, La Jolla, CA, USA). The kinetics of transfer of the fluorescent tracer to a cell somewhere in the middle of a filament (with two cell junctions) was quantified as previously described by calculating the recovery rate constant *R* from the formula C_B_ = C_0_ + C_R_ (1−e^−2*Rt*^), where C_B_ is fluorescence in the bleached cell, C_0_ is fluorescence immediately after the bleach and tending toward (C_0_ + C_R_) after fluorescence recovery, *t* is time and *R* is the recovery rate constant due to transfer of the tracer from neighboring cells (Merino-Puerto et al., 2011; Nürnberg et al., 2015; Nieves-Morión et al., 2017a).

## Results

### Inactivation of *amiC1* and *amiC2*

**Figure 1:**
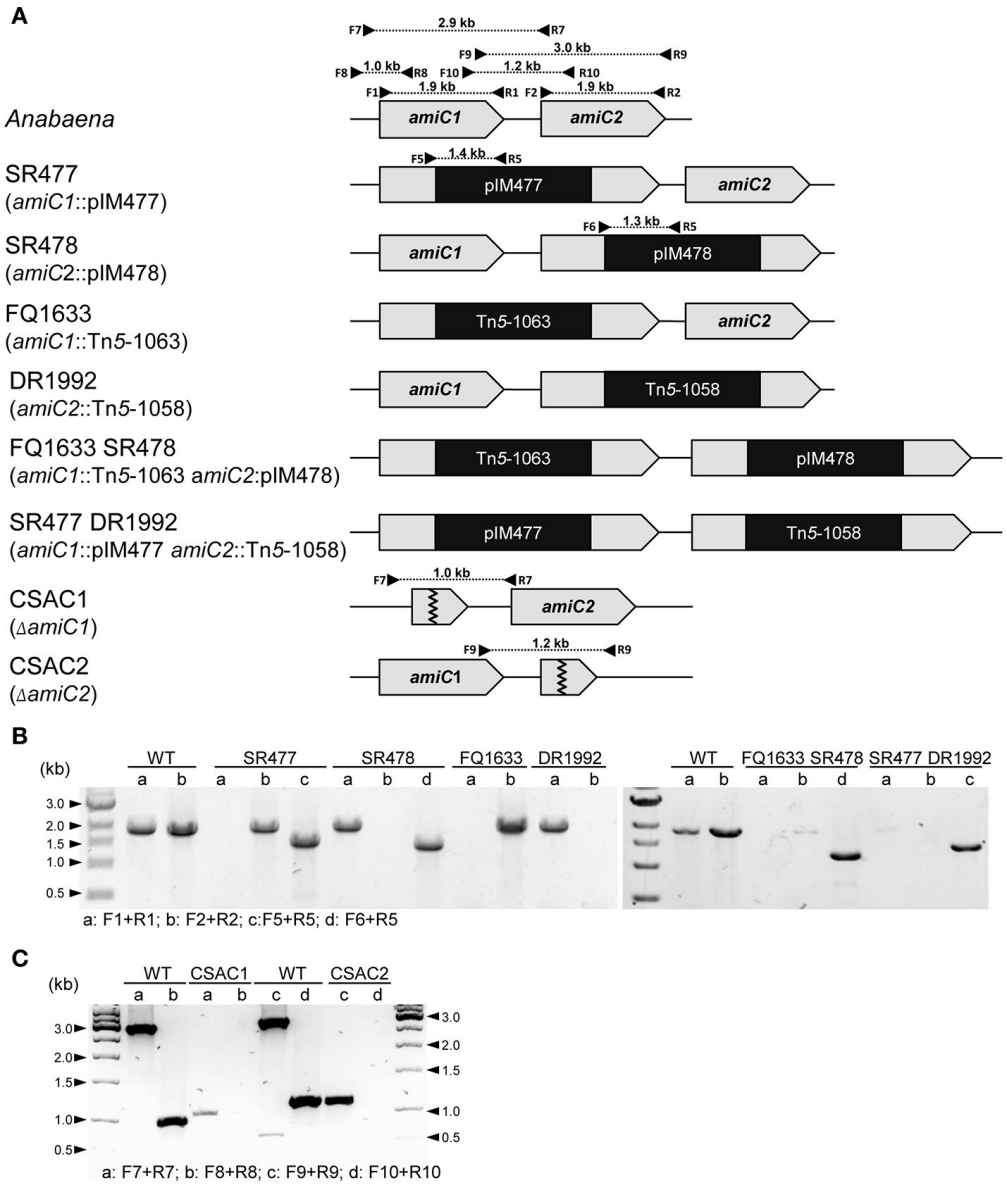
Structure of the genomic regions of *Anabaena* and various *amiC* mutant strains used in this work. **(A)** Schematic of the genomic region of *amiC1* and *amiC2* in *Anabaena* and indicated mutant strains. PCR primers and expected product sizes are indicated. **(B)** Analysis of the *amiC1* and *amiC2* inactivation in the indicated single and double mutants. Primer-pair a and b illustrate if wild-type *amiC1* and *amiC2* exist, respectively. Primer-pair c and d illustrate integration of pIM477 and pIM478 into the gene locus, respectively. **(C)** Analysis of the *amiC1* or *amiC2* deletion in the CSAC1 and CSAC2 mutants, respectively. Primer-pair a and c illustrate the existence of the respective wild-type genes and primer-pair b and d show the deletion of the respective genes. All primers are listed in Table S2.

**Figure 2:**
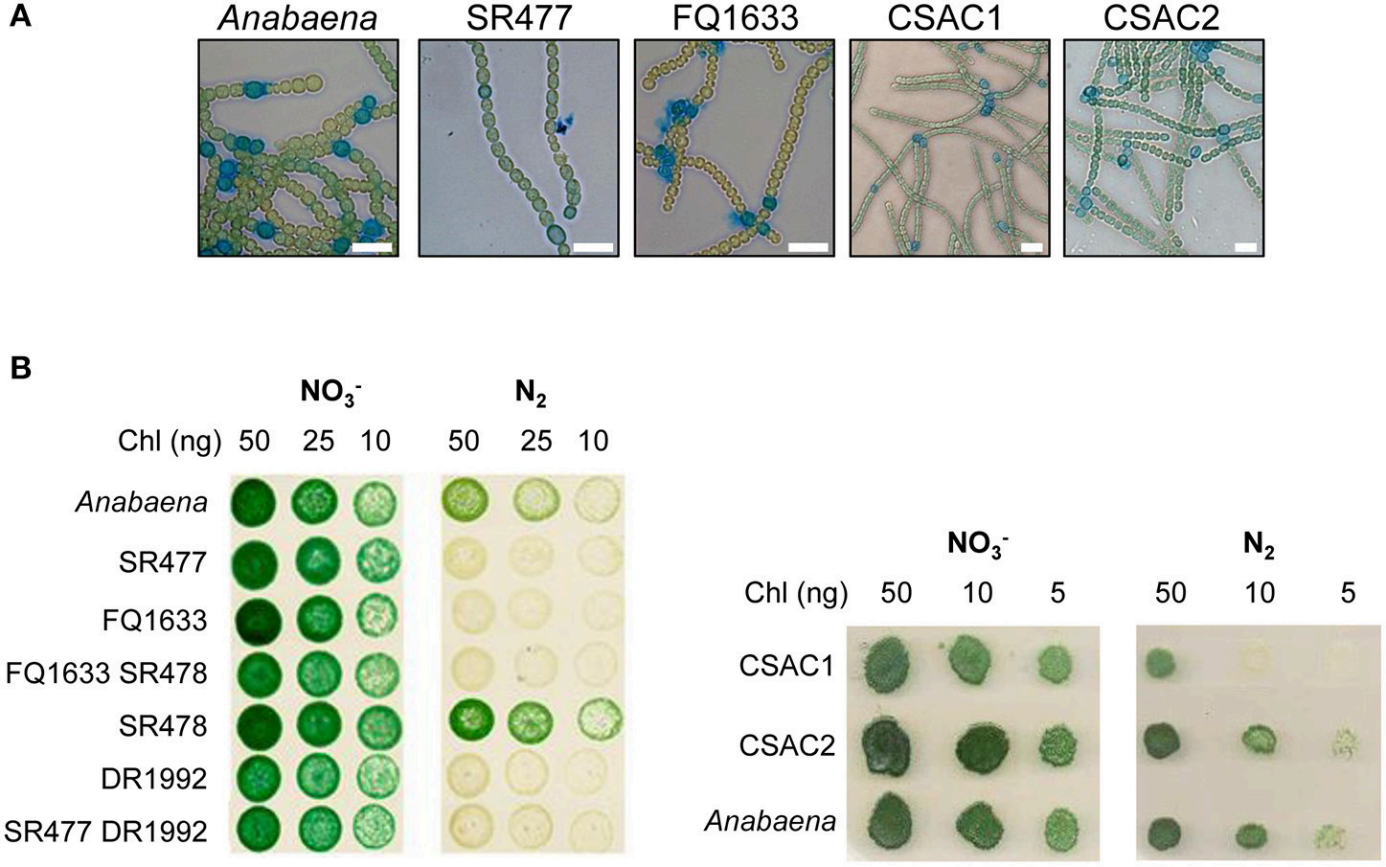
Morphology and growth of wild-type *Anabaena* and indicated mutant strains. **(A)** Alcian blue-stained filaments of indicated strains 48 h after nitrogen deprivation. Scale bars, 10 μm. **(B)** Growth of pseudocolonies on solid BG11 medium (${\text{NO}}_{3}^{-}$) and medium lacking a combined nitrogen source (N_2_). The plates were incubated under growth conditions for 7 (left side) and 6 (right side) days, respectively.

Since the phenotypes of already published *amiC2* mutants differed, one being Fox^−^ whereas the other was Fox^+^, and the phenotype of the published *amiC1* mutant SR477 was not stable (Zhu et al., 2001; Berendt et al., 2012), further mutants of *amiC1* and *amiC2* were generated by different approaches and their phenotypes were analyzed. In addition to previously described mutants SR477 (*amiC1*::pIM477) and SR478 (*amiC2*::pIM478; Berendt et al., 2012; reconstructed for this work), internally deleted mutants in *amiC1* (CSAC1) and *amiC2* (CSAC2), a transposon insertion mutant in *amiC1* (FQ1633) and a transposon insertion mutant in *amiC2* (DR1992; Zhu et al., 2001) were included in this study and characterized (Figure 1). PCR analysis detected no copies of the wild-type genes in the respective single mutants, indicating that they were fully segregated (Figure 1). As previously described, inactivation of *amiC1* led to a Fox^−^ phenotype (Berendt et al., 2012; Figure 2). Despite being Fox^−^, the newly created *amiC1* mutants CSAC1 and FQ1633 as well as the reconstructed mutant SR477 formed cells that were morphologically similar to heterocysts of the wild type (Figure 2). The newly created *amiC2* mutant CSAC2 showed a Fox^+^ phenotype as previously described for SR478 by Berendt et al. (2012).

**Figure 3:**
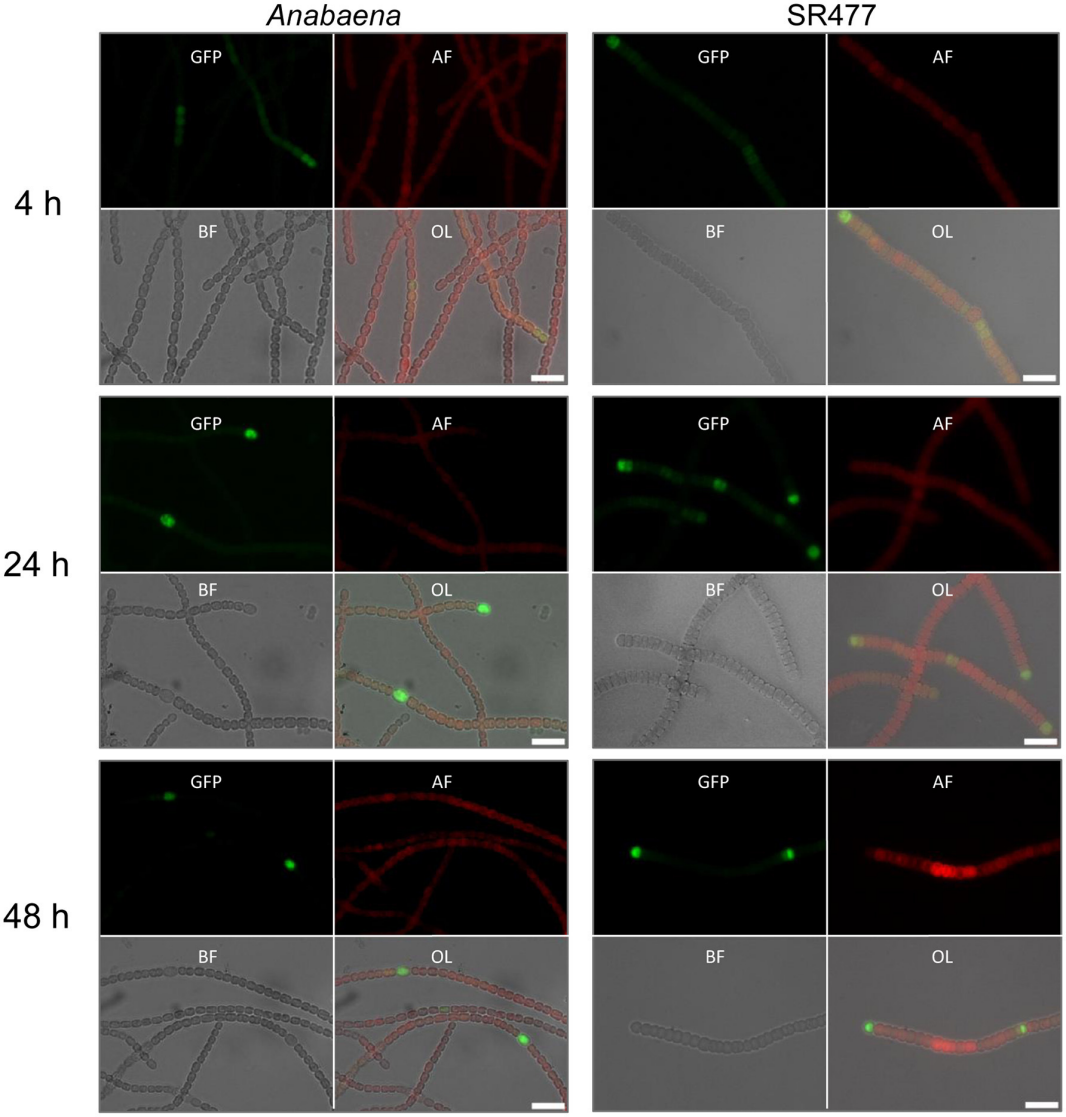
HetR promoter activity in *Anabaena* and *amiC1* mutant strain SR477. GFP expression under control of the *hetR* promoter in wild-type and SR477 background 4, 24, and 48 h after nitrogen step-down. Images of GFP-fluorescence (green; GFP), auto-fluorescence (red; AF), bright-field illumination (grayscale; BF), and an overlay (OL) are shown. Brightness and contrast were enhanced to improve visibility. Scale bars, 10 μm.

Heterocyst differentiation was delayed in the *amiC1* mutant strains compared to the wild-type strain. Since heterocyst differentiation depends on the early-induced heterocyst activator HetR (Black et al., 1993), we compared *hetR* expression in the *amiC1* mutant SR477 and in the wild-type strain by transferring the self-replicating plasmid pCSAM195 via conjugation to both strains. The plasmid encodes the *hetR* promoter transcriptionally fused to *e-gfp* (Alicia Muro-Pastor, unpublished) and allows analysis of the spatial and temporal activity of the *hetR* promoter by detection of GFP by fluorescence microscopy (Figure 3). GFP could be detected in clusters of cells in both the mutant and wild type after 4 h of nitrogen step-down, indicating that the *hetR* promoter was active. After 24 h, in the wild type, the expression of *e-gfp* (producing enhanced GFP) was clearly restricted to a single cell (a differentiated heterocyst) whereas in the SR477 mutant, GFP could be detected mostly in clusters of cells. After 48 h, the GFP signal was also restricted to single cells that might be considered heterocysts in the mutant.

To test whether the mutant heterocysts were functional and whether the Fox^−^ phenotype was caused by a defect in formation of a micro-oxic environment, we grew the filaments in BG11 medium, incubated them for 48 h in BG11_0_ medium, and measured acetylene reduction (Table 1). Compared to the wild type, acetylene reduction was diminished, but detectable, in all mutants, suggesting that the heterocysts were at least partially functional, producing micro-oxic conditions to allow nitrogenase activity.

**Table 1:** Nitrogenase activity under oxic conditions of wild-type and indicated mutant strains used in this work.

***Anabaena* strain**	**Nitrogenase Activity μmol ethylene (mg Chl)^−1^ h^−1^**
PCC 7120 (wild type)^*^	8.90 ± 0.72
SR477 (*amiC1*::pIM477)^*^	3.42 ± 0.52
FQ1633 (*amiC1*::Tn*5*-1063)^*^	3.64 ± 0.36
SR478 (*amiC2*::pIM478)^*^	4.18 ± 1.11
DR1992 (*amiC2*::Tn*5*-1058)^*^	3.65 ± 1.28
PCC 7120 (wild type)^**^	6.87 ± 1.15
CSAC1 (Δ*amiC1*)^**^	0.61 ± 0.07
CSAC2 (Δ*amiC2*)^**^	2.57 ± 1.20

*Filaments were incubated for 48 h in nitrogen-free medium before measuring nitrogenase activity by acetylene reduction assay*.

* *Strains were measured in the laboratory in Tübingen*.

** *Strains were measured in the laboratory in Seville*.

To assess whether the simultaneous inactivation of *amiC1* and *amiC2* has any additional effect compared to the single mutants, two double mutants were constructed: SR477 (*amiC1*::pIM477) DR1992 (*amiC2*::Tn*5*-1058) and FQ1633 (*amiC1*::Tn*5*-1063) SR478 (*amiC2*::pIM478; Figure 1). However, PCR analysis showed that faint bands corresponding to full-length copies of the wild-type genes *amiC1* and *amiC2* were still present in the double mutants even though the introduced vectors (SR477 and SR478) had recombined at the expected sites (Figure 1). Although full segregation of *amiC1* and *amiC2* was not achieved in either of the double mutants, which were therefore not complete knockout mutants, far more copies were present of the mutated than of the non-mutated *amiC* genes.

**Figure 4:**
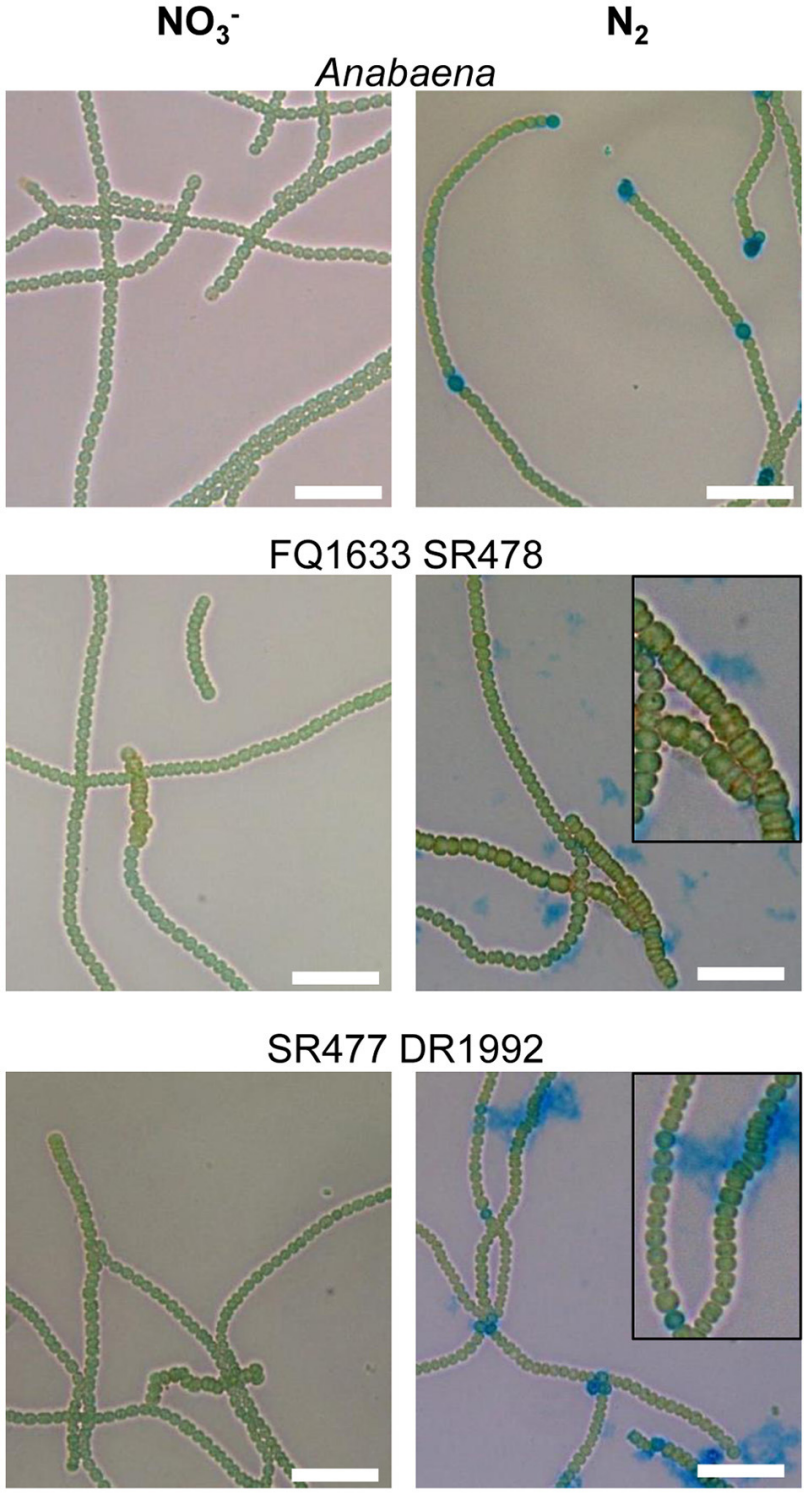
Filament morphology and differentiation of the *amiC1 amiC2* double mutants. Representative micrographs of *Anabaena* and the FQ1633 SR478 and SR477 DR1992 double mutants before (${\text{NO}}_{3}^{-}$) and 48 h after nitrogen step-down (N_2_) and Alcian blue staining are shown. Wild-type *Anabaena* showed Alcian blue-stained heterocysts after 2 days of nitrogen deprivation. The FQ1633 SR478 mutant, which is segregated in *amiC1* but not in *amiC2*, showed an aberrant phenotype (magnification) and did not show any heterocyst differentiation. The SR477 DR1992 mutant, which still has some *amiC1* wild-type copies, also showed an aberrant phenotype but was able to form heterocysts normally. Scale bars, 25 μm.

As with their single-mutant counterparts, the double mutants grew in medium with combined nitrogen, but failed to grow in nitrogen-free medium (Figure 2). With or without fixed nitrogen, the mutants showed an altered filament structure with two types of cells (Figure 4). Long, normally shaped filaments were formed by normally shaped vegetative cells and regularly placed septa. In addition, many filaments or segments of long filaments contained discoidal cells (i.e., cells whose length was shorter than their width), with aberrantly placed septa resulting in irregularly shaped filaments. Such was particularly the case for the FQ1633 SR478 mutant cells (Table S3). After nitrogen step-down, only the SR477 DR1992 mutant was able to form heterocysts as the wild type, and the envelope of the heterocysts could be stained with the polysaccharide-specific dye Alcian blue (Figure 4). In contrast, the FQ1633 SR478 mutant could not form heterocysts, even after 7 days of incubation in nitrogen-free medium (Figure S1). Very rarely, some single cells were stained with Alcian blue, but those cells were small, auto-fluorescent, and lacked typical polar bodies. Thus, the phenotype of the SR477 DR1992 double mutant was Fox^−^ Het^+^, whereas that of the FQ1633 SR478 double mutant may be considered Fox^−^ Het^−^.

### Mutations in *Anabaena amiC1* and *amiC2* led both to fewer nanopores and to impaired cell–cell communication

**Figure 5:**
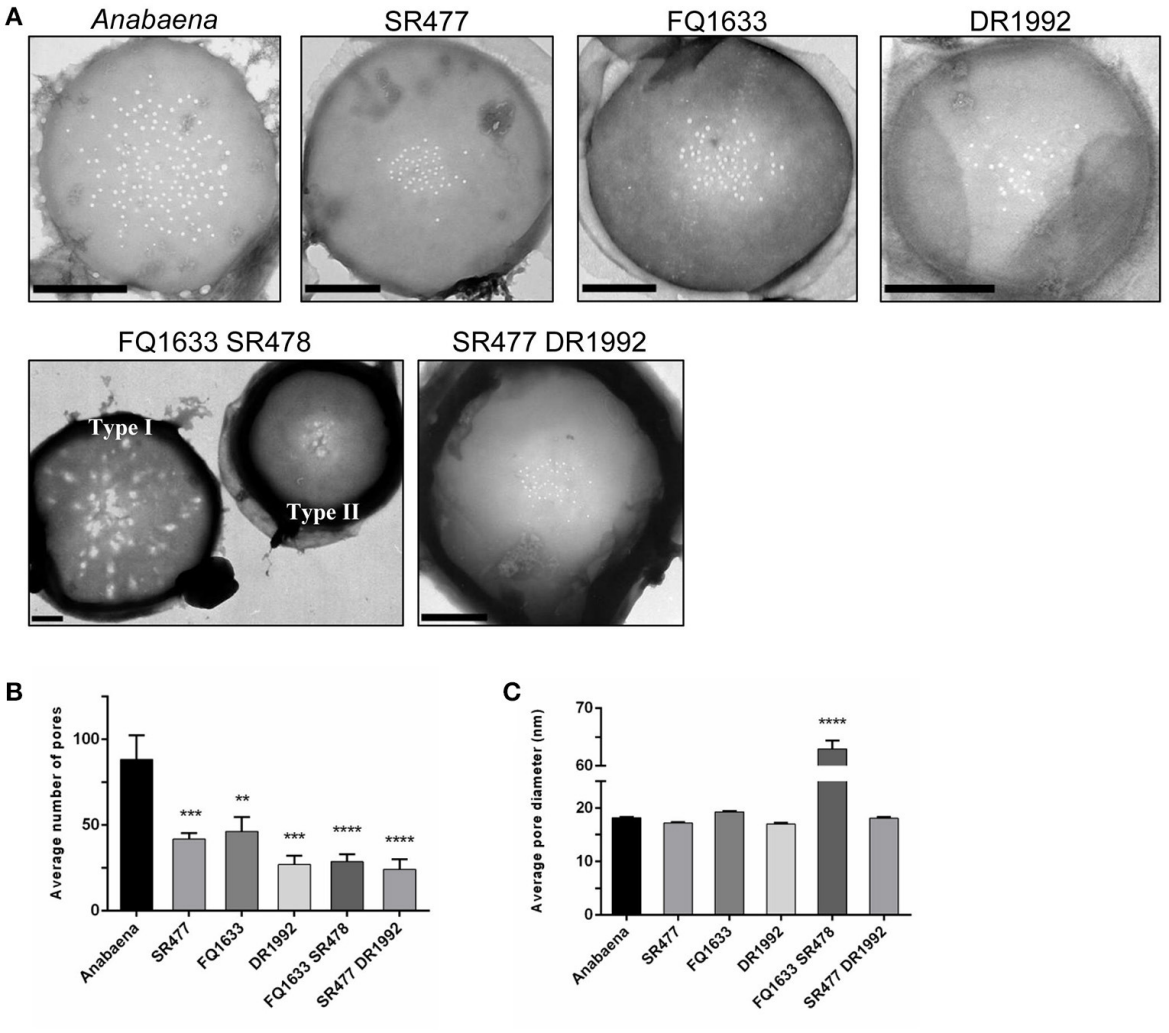
Septal nanopores in *Anabaena* and indicated *amiC* mutants. **(A)** Representative transmission electron micrographs of purified peptidoglycan sacculi from wild-type *Anabaena* and indicated mutant strains are shown. Scale bars, 500 nm. **(B)** The histogram shows the mean number of nanopores per septum ± *SD* of the mean for the different strains. Histogram **(C)** shows the average pore diameter ± *SD* of the mean for the indicated strains. Student's *t*-test (mutant vs. wild type) *P*-values are indicated as asterisks (^**^P ≤ 0.01; ^***^P ≤ 0.001; ^****^P ≤ 0.0001). See also Figure S2 and Table S4 for separate analysis depending on the type of nanopore array.

It was previously shown that the amidase AmiC2 is needed for nanopore array formation and cell–cell communication in *N. punctiforme* (Lehner et al., 2013). Here, we describe the nanopore formation in *amiC1* and *amiC2* mutants of *Anabaena*. We purified septal peptidoglycan of the various amidase mutants and analyzed nanopore array formation in disks of septal peptidoglycan by transmission electron microscopy (TEM; Figure 5A). Six to fourteen septa of each strain were analyzed.

Compared to the wild type, the mean number of nanopores per septum in all of the mutants shown was reduced (Figure 5B): the *amiC1* mutants SR477 and FQ1633 showed 47 and 52% of the count of nanopores, respectively; in the *amiC2* mutant DR1992, the quantity of nanopores was 31% of the wild type; and in the double mutants SR477 DR1992 and FQ1633 SR478, 27% and 33% of the wild type amount was measured, respectively. The diameters of the nanopores in the single *amiC* mutants and in the double mutant SR477 DR1992 did not differ significantly (Figure 5C). Interestingly, for the double mutant FQ1633 SR478, two types of aberrant nanopore arrays could be observed: one type had very large, irregularly shaped pores over the entire surface of the septum along with some normally shaped small pores (type I). In the other type (type II), an array of narrow nanopores in the center of the septal disks contained a mixture of much larger openings and regular nanopores (Figure S2, Table S4). The mean diameter of nanopores for this mutant was 63 ± 28 nm. Furthermore, the average diameter of the type I septal disks was much bigger compared to wild-type, type II or septal disks of the SR477 DR1992 mutant (Figure S2C).

**Figure 6:**
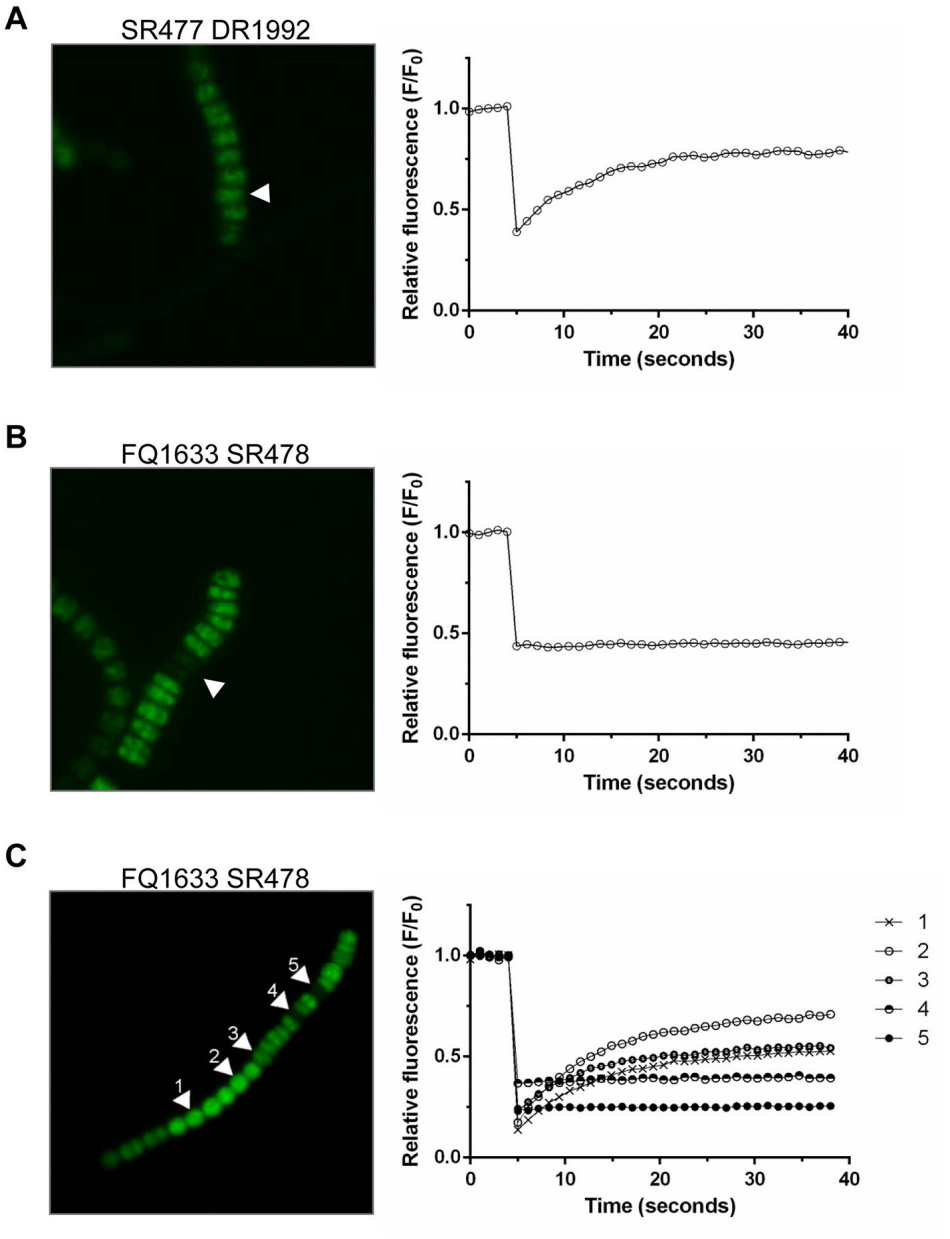
FRAP Analysis of aberrant filaments of the double mutants after being loaded with 5-CF. **(A)** Twenty-five seconds after being bleached, cells in an aberrant, double mutant SR477 DR1992 filament recovered (arrow, left). The corresponding graph shows the fluorescence recovery curve of the bleached cell in 1-second frames. **(B)** A similar bleach, but with an aberrant FQ1633 SR478 mutant filament, showed no recovery. **(C)** When filaments with morphologically normal and aberrant cells were analyzed, normal looking (cell 1 and 2) and the first cells in an aberrant segment (cell 3) were still able to communicate. Cells in the middle part of the aberrant segment showed no recovery (cell 4 and 5).

Because the different *amiC* mutants showed aberrant nanopores with reduced number and size, we investigated the intercellular exchange of calcein and 5-CF between vegetative cells of nitrate-grown cultures by FRAP analysis (Table 2). In the single mutants, a decreased rate of exchange of both calcein and 5-CF was observed, with rate constants about 60 and 30% of the wild-type activity, respectively. In the double mutants we could observe an interesting correlation between cell morphology and cell–cell communication. In the SR477 DR1992 mutant, recovery could be observed in aberrant-looking filaments with similar reduced rates as in the single mutants (Figure 6A). Interestingly, aberrant-looking filaments in the FQ1633 SR478 mutant showed no recovery in the bleached cells for either tracer (Figure 6B). When filaments of the FQ1633 SR478 mutant, which contained aberrant as well as normal-looking cells where analyzed, two different types of communication activities were observed: first, normal segments and the first cells in the aberrant segment showed reduced rates similar to single mutants. Second, recovery was completely abolished in the middle of aberrant segments (Figure 6C).

**Table 2:** Rate constants of fluorescent tracer exchange between vegetative cells in *Anabaena* and indicated *amiC* mutant strains used in this work.

	**Mean *R* (s^−1^) ± *SD* (*n*)**	
***Anabaena* strain**	**Calcein**	**5-CF**
PCC 7120	0.090 ± 0.018 (24)	0.120 ± 0.029 (12)
SR477	0.056 ± 0.013 (9)	0.038 ± 0.010 (33)
FQ1633	0.051 ± 0.015 (18)	0.032 ± 0.005 (3)
FQ1633 SR478		
(normal filaments)	0.057 ± 0.009 (5)	0.039 ± 0.012 (7)
(aberrant filaments)^*^	0.000 ± 0.000 (6)	0.000 ± 0.000 (16)
SR477 DR1992	0.047 ± 0.008 (2)	0.051 ± 0.002 (2)

*FRAP analysis was performed and evaluated as described in Materials and Methods*.

*Data are mean ± SD from the results obtained with the number n of filaments subjected to FRAP analysis in parenthesis*.

* *39% of cells showed aberrant morphology (see also Table S3)*.

### Mutation of *amiC1* in *sepJ* and *fraCD* mutant backgrounds diminishes fragmentation

**Figure 7:**
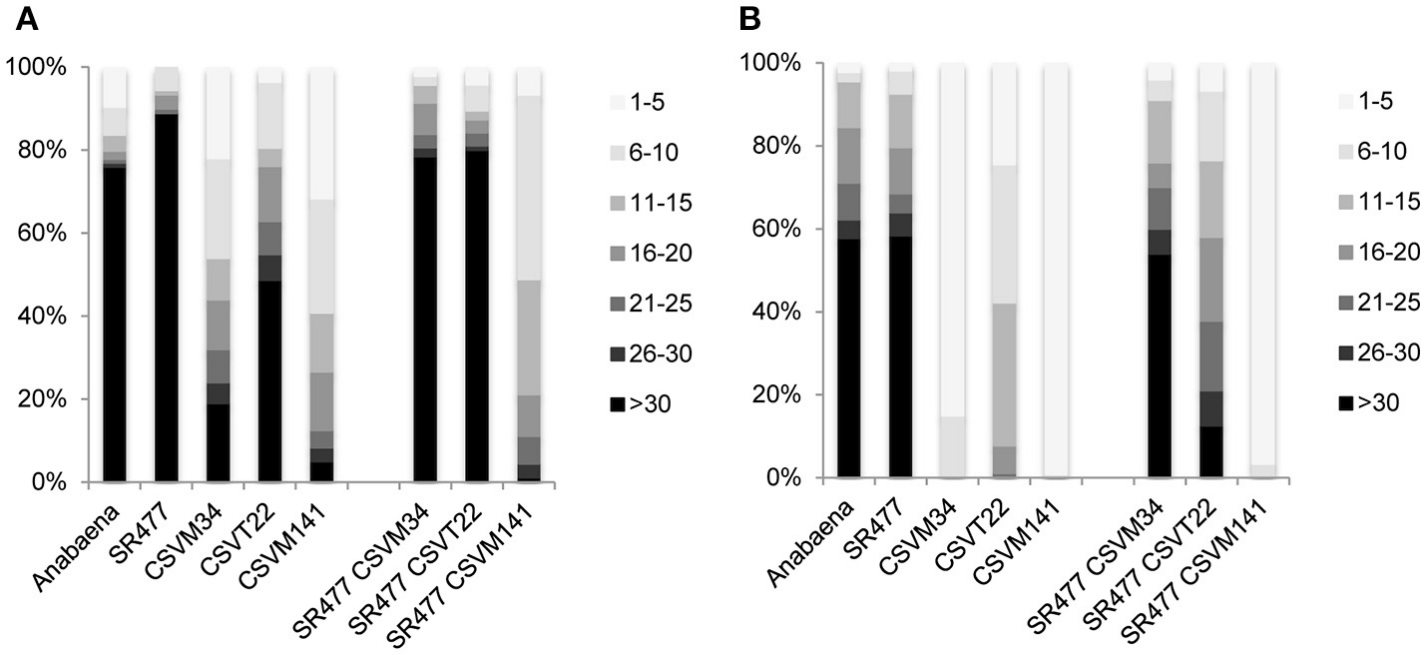
Filament fragmentation of *Anabaena* wild type and indicated mutants used in this work. Samples of shaken cultures grown in BG11 medium **(A)** and incubated under the same culture conditions for 48 h in BG11_0_ medium **(B)** were taken with great care to prevent disruption. A total of 90–120 filaments were counted for each strain and culture condition and ascribed to the size intervals indicated on the right. Filament size is expressed as cells per filament, the gray scale code is on the right and the percentages of filaments are indicated on the left.

*Anabaena* Δ*sepJ* (CSVM34), Δ*fraC* Δ*fraD* (CSVT22), and Δ*sepJ* Δ*fraC* Δ*fraD* (CSVM141) mutants have been previously characterized and showed a severe filament fragmentation phenotype (Mariscal et al., 2011; Merino-Puerto et al., 2011; Nürnberg et al., 2015). To test whether the inactivation of *amiC1* in these mutants has an effect on fragmentation, mutants were created by transfer of the *amiC1*-inactivating construct pIM477 (Berendt et al., 2012) to strain CSVM34, double mutant strain CSVT22 and triple mutant strain CSVM141. The resulting strains SR477 CSVM34, SR477 CSVT22, and SR477 CSVM141 lacked wild-type copies of the genes as confirmed by PCR (Figure S3). Filament fragmentation was investigated in cultures treated as previously described (Merino-Puerto et al., 2010) and incubated with or without combined nitrogen. In this study, the quadruple mutant SR477 CSVM141 still formed extremely short filaments, whereas the double mutant SR477 CSVM34 and the triple mutant SR477 CSVT22 showed a weaker fragmentation phenotype (i.e., longer filaments) than the respective mutants without additional *amiC1* inactivation (Figure 7). Furthermore, the effect of stabilizing the filament against fragmentation was stronger in the Δ*sepJ* mutant background than in the Δ*fraC* Δ*fraD* mutant background.

### Mutation of *amiC1* influences SepJ localization

**Figure 8:**
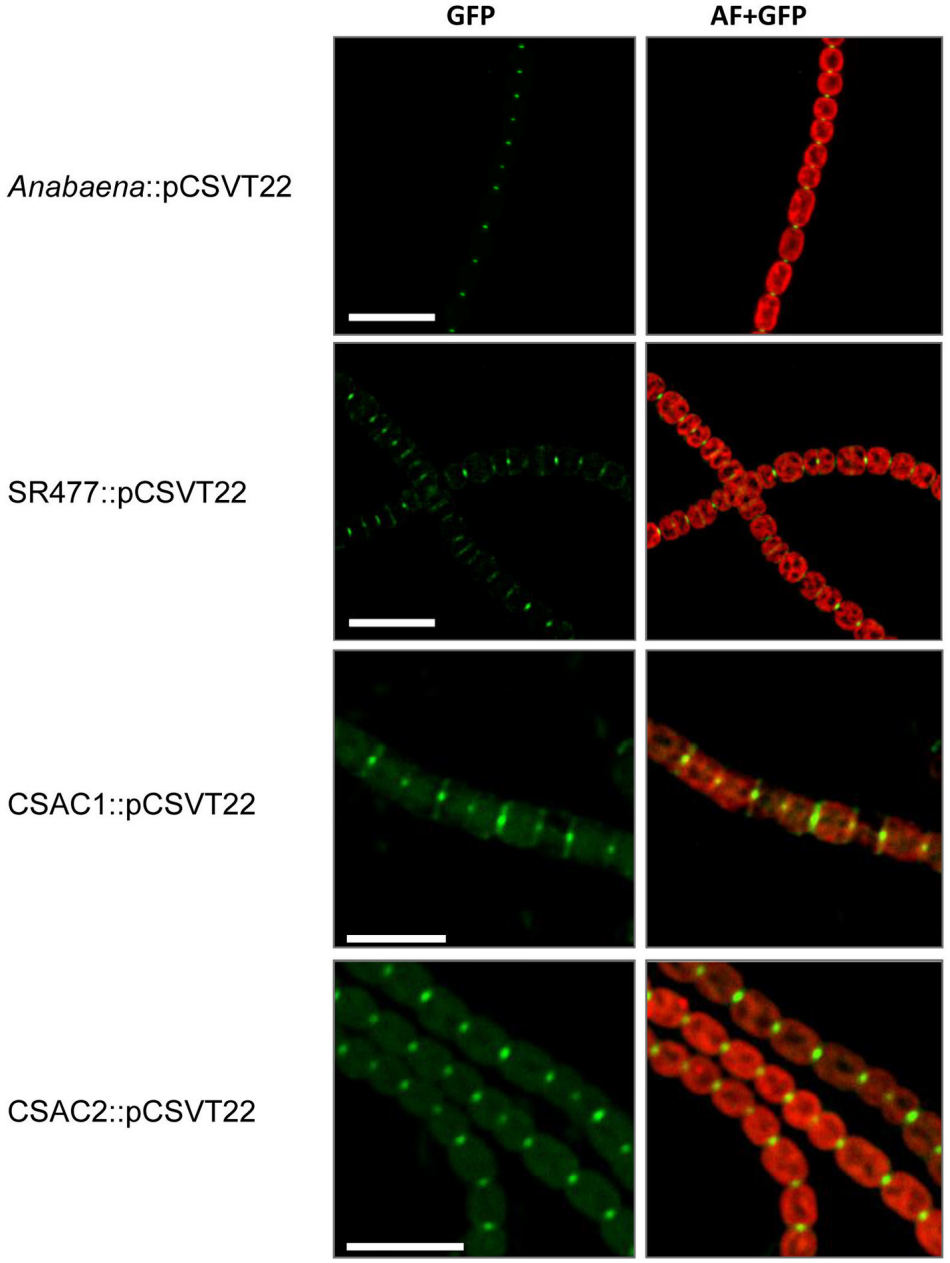
Subcellular localization of SepJ-GFP. Subcellular localization of SepJ-GFP in *Anabaena*::pCSVT22, the *amiC1* mutant strain SR477::pCSVT22, the Δ*amiC1* mutant CSAC1::pCSVT22 and the Δ*amiC*2 CSAC2::pCSVT22 visualized by fluorescence microscopy. The images show GFP-fluorescence (green; GFP) and an overlay of the GFP-fluorescence with the auto-fluorescence (red; AF). Brightness and contrast were enhanced to improve visibility. Scale bars, 10 μm.

Because inactivation of *amiC1* drastically diminished the fragmentation phenotype of the *sepJ* mutant, some type of interaction between AmiC1 and SepJ is possible. We therefore asked whether the absence of AmiC1 influences SepJ localization. We studied the subcellular localization of SepJ-GFP in the *amiC1* mutants SR477 and CSAC1 and in the *amiC2* mutant CSAC2 by transferring to these strains plasmid pCSVT22 (Merino-Puerto et al., 2010), which bears a translational *sepJ-gfp* fusion, resulting in strains SR477::pCSVT22, CSAC1::pCSVT22 and CSAC2::pCSVT22, respectively. The plasmid was also transferred to the wild type as control. Strains with inactivated *amiC1* showed a strong effect on SepJ-GFP localization (Figure 8). In the wild type, SepJ-GFP regularly localized to the center of each septum as a focal spot as described previously (Flores et al., 2007). However, in mutants lacking *amiC1*, SepJ-GFP was less focused and often a broad GFP signal could be observed over the entire septum. In contrast, deletion of *amiC2* had no impact on the localization of SepJ-GFP (Figure 8). These results indicate that AmiC1 but not AmiC2 appears to be needed for proper subcellular localization of SepJ at the center of intercellular septa.

## Discussion

In recent years, cell–cell communication has been recognized as an important character of unicellular bacteria living in communities such as biofilms (Hooshangi and Bentley, 2008; Prindle et al., 2015; Asfahl and Schuster, 2017). Whereas in these bacterial populations cells show a multicellular-like coordinated behavior, *Anabaena* and other heterocyst-forming cyanobacteria are true multicellular organisms with elaborated communication along the filament (Mullineaux et al., 2008; Herrero et al., 2016). For patterned formation of heterocysts, signal migration along the filament is essential to ensure proper distribution of nutrients. Besides signaling molecules such as the small inhibitory PatS peptide, metabolites have to be exchanged between cells. Heterocysts obtain, from vegetative cells, reduced carbon in the form of -at least- sucrose and glutamate, the substrate of glutamine synthetase. In return, heterocysts deliver fixed nitrogen compounds, presumably including glutamine and β-aspartyl-arginine (Herrero et al., 2016). How exchange and communication occurs is currently under intensive investigation (Lehner et al., 2013; Nürnberg et al., 2014, 2015; Büttner et al., 2016; Nieves-Morión et al., 2017a,b; Zheng et al., 2017).

It has been shown that the amidase AmiC2 from *N. punctiforme* perforates the septal peptidoglycan creating an array of nanopores, which appear to be essential for filament morphology, intercellular communication, and cell differentiation (Lehner et al., 2011). That amidase is one of two proteins, AmiC1 and AmiC2, that are conserved in heterocyst-forming cyanobacteria. Whereas, mutation of *amiC1* in *N. punctiforme* was not achieved, the genes homologous to *amiC1* and *amiC2* have been functionally characterized in *Anabaena* (Zhu et al., 2001; Berendt et al., 2012).

To gain deeper insight into the function of AmiC-related amidases in cell–cell communication and cellular differentiation in *Anabaena*, further mutants in *amiC1* and *amiC2* were generated. Such mutants in *amiC1* did not grow diazotrophically, whereas the Δ*amiC2* mutant CSAC2 showed no Fox^−^ phenotype under the tested growth conditions, confirming the report by Berendt et al. (2012). Heterocysts produced in the *amiC1* mutants SR477, CSAC1 and FQ1633 showed reduced but significant nitrogenase activity under oxic conditions, indicating that in principle, functional heterocysts were produced. Presumably, the fixed nitrogen was not delivered to the vegetative cells of the filament, preventing them from growing. Alternatively, the lower activity of nitrogenase could be due to a lack of reduced carbon delivered to the heterocysts from neighboring vegetative cells. It is also possible that the low nitrogenase activity in the mutants could be due to defects in the heterocyst envelope, which is known to protect nitrogenase from external oxygen. As suggested earlier (Zhu et al., 2001; Léganes et al., 2005), modification of the peptidoglycan is a prerequisite for proper heterocyst envelope formation. However, Alcian blue staining showed the presence of the heterocyst polysaccharide layer in the mutant heterocysts.

It was shown previously that in *N. punctiforme*, an *amiC2* mutant completely lacked nanopores in the septal peptidoglycan (Lehner et al., 2011, 2013). To investigate the role of the AmiC homologs in *Anabaena*, sacculi of the *amiC1* and *amiC2* mutants were analyzed by TEM. Although the nanopore arrays were diminished, nanopores were still present. The fact that a reduction in nanopore count is accompanied by a reduction of intercellular molecular exchange corroborates the importance of nanopores for septal junction formation and cell–cell communication. Although 5-CF is smaller than calcein, exchange of 5-CF was slower than calcein exchange in the *amiC* mutants, confirming that size is not the only determining property. The greater negative charge of calcein could facilitate its passage through whatever conduits remain. The amidases seem to be specifically related to certain junction complexes, having less influence on formation of other septal junctions. Although mutants SR477, FQ1633 and DR1992 have a substantial nitrogenase activity (Table 1), they fail to grow on N_2_ (Figure 2), implying that the transfer of products of nitrogen fixation from heterocysts to vegetative cells is affected, due presumably to a reduced number of septal junction complexes involved in the transfer of amino acids (and 5-CF). This would also explain the normal growth of the mutants on nitrate, when such cell–cell exchange of metabolites is not needed.

In a fully segregated *amiC1* mutant, a complete knockout of *amiC2* was not achieved, and *vice versa*, suggesting that at least one of the two enzymes is required for normal growth and cell division. In contrast to the single mutants, and in line with the septal localization of the amidases in *Anabaena* and *N. punctiforme* (Lehner et al., 2011, 2013; Berendt et al., 2012; Büttner et al., 2016; Faulhaber, 2017), cell and filament morphology was affected in both kinds of double mutants, suggesting that AmiC-type amidases are involved also in cell division and in correct placement of the septal plane. In the fully segregated *amiC1* mutant, heterocyst differentiation was completely inhibited when pIM478 appears to have inserted in most of the multiple chromosomal copies of strain FQ1633 SR478. This shows that a low copy number of *amiC2* is insufficient for differentiation. In the same mutant strain, tracer exchange was not detected in filaments with an aberrant phenotype. However, the mutant with deletion of *amiC1* in an *amiC2* mutant background (SR477 DR1992) was able to differentiate heterocysts and showed cell–cell communication also in filaments with an aberrant phenotype. Because *Anabaena* cells have multiple copies of their genome, the filaments with apparently unaffected morphology and molecular exchange may arise from cells with an enhanced *amiC1* copy number, whereas the filaments with aberrant morphology and impaired intercellular transfer might contain cells with few or no residual copies of *amiC1*.

In line with the two types of filament morphology, we observed two types of nanopore arrays in purified peptidoglycan samples of the double mutant FQ1633 SR478. We suggest that the type I septa with widely dispersed, abnormally large oval pores represent impaired cell–cell communication machinery that may have resulted both in (i) the inability of cells to become heterocysts and (ii) filaments with aberrant morphology. We conclude that the aberrant, non-communicating filaments in the FQ1633 SR478 mutant may have lost all of their *amiC2* copies, resulting in a type I nanopore array and a loss of cell–cell communication. Full segregation in the double mutant would probably lead to cell death. The delay of the *hetR* promoter in the *amiC1* mutant strain SR477 also suggests the importance for heterocyst differentiation of communication occurring through direct cell–cell joining complexes. Recently a third AmiC-like protein (All1140, AmiC3) has been described as being also involved in heterocyst differentiation and intercellular molecular exchange (Zheng et al., 2017).

With respect to the nanopore array in the *amiC1 amiC2* double mutants, decreasing the level of AmiC1 in the *amiC2* mutant background did not drastically reduce the amount of nanopores in the septa, suggesting that the remaining level of AmiC1 sufficed to form a small nanopore array for requisite cell–cell communication. Reducing the level of AmiC2 in the *amiC1* mutant background severely affected the shape of the nanopores, suggesting that AmiC2 is involved in normal formation of the nanopore array. It is possible that AmiC2 interacts with proteins, such as SjcF1 (Rudolf et al., 2015), that are necessary for correct shaping of nanopores. Our data illustrate the importance of AmiC1 and AmiC2 in the formation of arrays of nanopores, although these amidases appear to be redundant to some extent, as is the case in *E. coli* (Heidrich et al., 2002) and as previously suggested by Berendt et al. (2012). Nonetheless, AmiC1 seems to be the more important amidase, because inactivation of *amiC1* shows a stronger phenotype: inactivation of *amiC2* can be compensated by *amiC1*, whereas *amiC2* seems unable to compensate for loss or reduction of *amiC1*. In addition, overexpression of *amiC1* under a copper-inducible promoter led, in contrast to *amiC2*, to lysis of the cells (Berendt et al., 2012).

**Figure 9:**
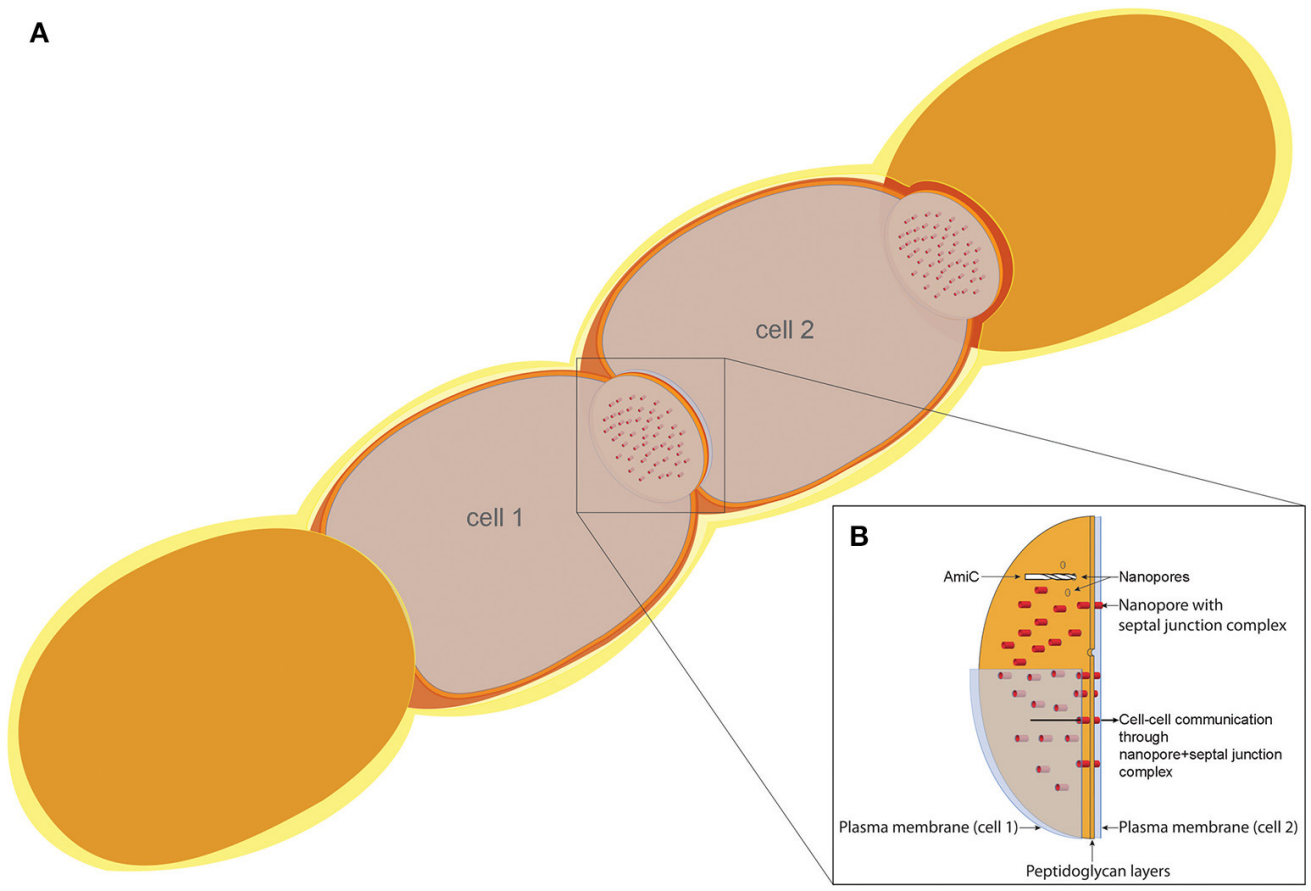
Model of the cell–cell communication structure in filamentous cyanobacteria. **(A)** Schematic filament of vegetative *Anabaena* cells with two sectioned cells (cell 1 and cell 2), allowing the view on top of the septal disks. **(B)** Model of the septal disks between vegetative cells, showing AmiC drilling a nanopore, thereby forming the nanopore array. Nanopores containing septal junction complexes allow the exchange of molecules through the septal peptidoglycan.

Since we have observed a delocalization of SepJ in two different *amiC1* mutant strains, and inactivation of *amiC1* counteracts the fragmentation phenotypes of *sepJ* and *fraC/fraD* mutants, a functional relation between AmiC1 and SepJ on one hand, and of AmiC1 and FraCD on the other, is evident. Previously, we showed that in the *sepJ* and *fraCD* mutants, the number of septal nanopores is more drastically reduced compared to the amidase mutants (Nürnberg et al., 2015), and that overexpression of SepJ increases the number of septal nanopores (Mariscal et al., 2016). These results imply that the generation of septal junctions by SepJ and FraCD might be directly coupled to nanopore formation. AmiC1 is a cell wall lytic, *N*-acetylmuramyl-L-alanine amidase that appears to digest nanopores in septal peptidoglycan, thereby providing for “cell–cell communication,” i.e., transfer of calcein or 5-CF between cells, especially between vegetative cells and heterocysts. The structure and localization of SepJ suggests that it traverses septa. It could be that SepJ and FraCD locate where nanopores will be built and direct AmiC proteins to digest the nanopores at specific sites, and that SepJ-septal junctions merely occupy available nanopores (Figure 9).

In summary, the collective data, especially the findings of septal ultrastructure and exchange of molecules between cells of mutants lacking *amiC1, amiC2*, or both genes, strengthen the hypothesis that the two amidases have related functions in *Anabaena*. They are required for modeling of the septal peptidoglycan, which is essential for intercellular molecular exchange and necessary for the development of a heterocyst-containing filament, and thus play a fundamental role in multicellularity. To attain their function fully, the amidases somehow allow septal junctions based on SepJ (and possibly also FraCD) to coordinate and build fully functional intercellular septa.

## Author contributions

JB: designed and performed experiments, interpreted data, wrote most of the manuscript, drafted the work, made manuscript revisions and gave final approval. He made substantial contributions to the design of the work and the analysis or interpretation of data for the work and agreed to be accountable for all aspects of the work in ensuring that questions related to the accuracy or integrity of any part of the work were appropriately investigated and resolved. AC: performed experiments, interpreted data, drafted figures and tables, approved the manuscript. QF: designed and performed experiments, revised the manuscript critically, approved the version to be published and agreed to be accountable for all aspects of the work in ensuring that questions related to the accuracy or integrity of any part of the work were appropriately investigated and resolved. EF: designed research, interpreted data, revised the work critically for important intellectual content, and made manuscript revisions. He agreed to be accountable for all aspects of the work in ensuring that questions related to the accuracy or integrity of any part of the work were appropriately investigated and resolved. KF: designed research, interpreted data, made substantial contributions to the design of the work; made acquisition, analysis and interpretation of data for the work; made manuscript revisions; revised the work critically for important intellectual content and made final approval; and agreed to be accountable for all aspects of the work in ensuring that questions related to the accuracy or integrity of any part of the work were appropriately investigated and resolved. VM: designed and supervised the research in Seville; interpreted data; made substantial contributions to the design of the work; made acquisition, analysis and interpretation of data for the work; made manuscript revisions; and agreed to be accountable for all aspects of the work in ensuring that questions related to the accuracy or integrity of any part of the work were appropriately investigated and resolved. CM: provided training in FRAP, analysis and interpretation of data; critically read and approved the manuscript; and agreed to be accountable for all aspects of the work in ensuring that questions related to the accuracy or integrity of any part of the work were appropriately investigated and resolved. RP: designed and performed experiments, interpreted data, and approved the manuscript. She agreed to be accountable for all aspects of the work in ensuring that questions related to the accuracy or integrity of any part of the work were appropriately investigated and resolved. NS: performed experiments, interpreted data, made figures, and approved the manuscript. CW: made acquisitions and analysis of data for the work, revised the work critically for important intellectual content, made final approval and agreed to be accountable for all aspects of the work in ensuring that questions related to the accuracy or integrity of any part of the work were appropriately investigated and resolved. IM: designed and supervised the research in Tübingen, performed experiments, wrote part of the manuscript, made substantial contributions to the design of the work and made acquisition, analysis and interpretation of data for the work. She revised the work critically for important intellectual content and made final approval, made manuscript revisions, and agreed to be accountable for all aspects of the work in ensuring that questions related to the accuracy or integrity of any part of the work were appropriately investigated and resolved.

### Conflict of interest statement

The authors declare that the research was conducted in the absence of any commercial or financial relationships that could be construed as a potential conflict of interest.
