# Supplementary material for: Role of Two Cell Wall Amidases in Septal Junction and Nanopore Formation in the Multicellular Cyanobacterium Anabaena sp. PCC 7120
Source: Front Cell Infect Microbiol. 2017 Sep 5;7:386. doi: 10.3389/fcimb.2017.00386 (PMC5591844; doi:10.3389/fcimb.2017.00386)
Supplement: Supplementary file 1 [file Presentation1.pdf]

Supplemental material

Role of Two Cell Wall Amidases in Septal Junction and Nanopore Formation in The Multicellular Cyanobacterium *Anabaena* sp. PCC 7120

Jan Bornikoel[1], Alejandro Carrión[2], Qing Fan[5], Enrique Flores[2], Karl Forchhammer[1], Vicente Mariscal[2], Conrad W. Mullineaux[4], Rebeca Perez[1], Nadine Silber[1], C. Peter Wolk[3], Iris Maldener[1*]

***Correspondence:** iris.maldener@uni-tuebingen.de

[1] IMIT Organismic Interactions, University of Tübingen, Tübingen, Germany
[2] Instituto de Bioquímica Vegetal y Fotosíntesis, Consejo Superior de Investigaciones Científicas and Universidad de Sevilla, Seville, Spain
[3] MSU-DOE Plant Research Laboratory and Department of Plant Biology, Michigan State University, East Lansing, MI, USA
[4] School of Biological and Chemical Sciences, Queen Mary University of London, London, United Kingdom
[5] Department of Microbiology-Immunology, Feinberg School of Medicine of Northwestern University, Chicago, IL, USA

Table S1

Strains and plasmids used in this work.

Strain or plasmid	Derivation and/or relevant characteristics	Source or reference
Anabaena sp. strains		
PCC 7120	wild-type	
Anabaena::pCSVT22	SepJ-GFP, Nm^r	Merino-Puerto *et al.*, 2010
CSAC1	Δ*amiC1*	This study
CSAC1::pCSVT22	Δ*amiC1*, SepJ-GFP, Nm^r	This study
CSAC2	Δ*amiC2*	This study
CSAC2::pCSVT22	Δ*amiC2*, SepJ-GFP, Nm^r	This study
CSVM141	Δ*sepJ* Δ*fraC* Δ*fraD*	Nürnberg *et al.*, 2015
CSVM34	Δ*sepJ*	Mariscal *et al.*, 2011
CSVT22	Δ*fraC* Δ*fraD*	Merino-Puerto *et al.*, 2011
DR1992	*amiC2*::Tn*5*-1058, Nm^r, Sm^r, Bm^r (Bleomycin)	Zhu *et al.*, 2001
FQ1633	*amiC1*::Tn*5*-1063, Nm^r, Sm^r, Bm^r	This study
FQ1633 SR478	*amiC1*::Tn*5*-1063, *amiC2*::pIM478, Nm^r, Sm^r, Sp^r, Bm^r	This study
SR477	*amiC1*::pIM477, Sm^r, Sp^r	Berendt *et al.*, 2012
SR477 CSVM141	*amiC1*::pIM477, Δ*sepJ* Δ*fraC* Δ*fraD*, Sm^r, Sp^r	This study
SR477 CSVM34	*amiC1*::pIM477, Δ*sepJ*, Sm^r, Sp^r	This study
SR477 CSVT22	*amiC1*::pIM477, Δ*fraC* Δ*fraD*, Sm^r, Sp^r	This study
SR477 DR1992	*amiC1*::pIM477, *amiC2*::Tn*5*-1058, Nm^r, Sm^r, Sp^r, Bm^r	This study
SR477::pCSVT22	*amiC1*::pIM477, SepJ-GFP, Nm^r, Sm^r, Sp^r	This study
SR478	*amiC2*::pIM478, Sm^r, Sp^r	Berendt *et al.*, 2012
Plasmid		
pCSAC1	Flanking regions of *amiC1* fused and cloned in pSpark	This study
pCSAC3	SacI fragment from pCSAC1 cloned in pCSRO	This study
pCSAC10	Flanking regions of *amiC2* fused and cloned in pSpark	This study
pCSAC12	SacI fragment from pCSAC10 cloned in pCSRO	This study
pCSAM195	*PhetR*::*gfp*, Nm^R	Alicia Muro-Pastor, unpublished
pCSVT22	*sepJ-gfp* from pCSAM135 cloned in pRL424; Nm^R	Elhai and Wolk, 1988b; Merino-Puerto *et al.*, 2010
pIM477	Internal fragment of *amiC1* in pRL277, Sm^r, Sp^r	Black *et al.*, 1993; Berendt *et al.*, 2012
pIM478	Internal fragment of *amiC2* in pRL277, Sm^r, Sp^r	Black *et al.*, 1993; Berendt *et al.*, 2012

Table S2

Oligonucleotides used in this work in PCR.

Primer name	Sequence (5‘ to 3‘)
F1	AGATCTGTGAAATTACACTGGTTACTATCTGG
F2	AGATCTGTGAAATTACACTGGTTACTATCCG
F3	ATACAGGAAATTAGAGTGAGC
F4	TGCCGTTCCTTGTCATCT
F5	CTCGAGCAGTGGTGGCAATCCTCAAG
F6	CTCGAGTTACGAGTTACAGGCGATGG
F7	GAGCTCGTATTCGGAATGTTATCTGTTG
F8	GATTGGCGTGGGGATGATT
F9	GAGCTCAAACTTACTATTACGATAGCGG
F10	CTCGCTACCACACCTCCCTTAC
F11	TAATAAGGATCCGTATTCTTCAGTACCTCAAACG
F12	TAATAAGGATCCATCCTGCAATATTTAAAGAGAC
R1	CTGCAGCTATCGTTTGAGGTACTGAAG
R2	CTGCAGTTACTGTCTCTTTAAATATTGCAGGATACC
R3	TGAGCCAGAAGTCCAGAG
R4	GGATCCAGTACGGACGATAAA
R5	ACCTATCTCAGCGATCTGTC
R6	CAAGAATTGGGACAACTCC
R7	GAGCTCGTAAATCAATAACTAAACGTGTC
R8	AGGCTGGCGACGATAGTAATAAG
R9	GAGCTCATACGCTTACCTTGCTGTAC
R10	ACCGCCTATTTGCTGTGTTGA
R11	GGATCCTTATTATGTACCAGATAGTAACCAGTGTA
R12	GGATCCTTATTAGGATAGTAACCAGTGTAATTTCAC

Table S3

Frequencies of aberrant cells in the *amiC1 amiC2* double mutants. Filaments were grown in BG11 medium in the presence of 5 µg ml^{-1} Sm, 5µg ml^{-1} Sp and 50 µg ml^{-1} Nm. After reaching an OD_{750} of 0.5, samples were taken with great care to prevent disruption. Cells of a total of 160 filaments were counted for each strain and categorized as normal or aberrant based on cell morphology.

Anabaena strain	Aberrant cells	Total cells count	Normal filaments	Aberrant filaments (only containing aberrant cells)	Normal filaments with aberrant segments
FQ1633 SR478	39 %	3970	31 %	28 %	41 %
SR477 DR1992	24 %	5265	53 %	20 %	27 %

Table S4

Statistical analysis of nanopore diameter distribution. See also figure S2.

Strain	Nanopores analyzed	Average diameter (nm)		P-value of mean difference					
		Mean (SEM)	Median (25%/75%)	SR477	FQ1633	DR1992	FQ1633 SR478 Type I	FQ1633 SR478 Type II	SR477 DR1992
Anabaena	1061	18 (0.14)	17.6 (14/22)	ns	ns	ns	≤ 0.0001	≤ 0.0001	ns
SR477	585	17 (0.15)	17.4 (15/20)	-	≤ 0.05	ns	≤ 0.0001	≤ 0.0001	ns
FQ1633	430	19 (0.16)	18.9 (17/22)	-	-	ns	≤ 0.0001	≤ 0.0001	ns
DR1992	162	17 (0.22)	17.0 (15/19)	-	-	-	≤ 0.0001	≤ 0.0001	ns
FQ1633 SR478 Type I	200	76 (1.84)	71.7 (57/91)	-	-	-	-	≤ 0.0001	≤ 0.0001
FQ1633 SR478 Type II	174	48 (1.81)	43.3 (32/59)	-	-	-	-	-	≤ 0.0001
SR477 DR1992	204	18 (0.27)	17.7 (16/20)	-	-	-	-	-	-

Figure S1

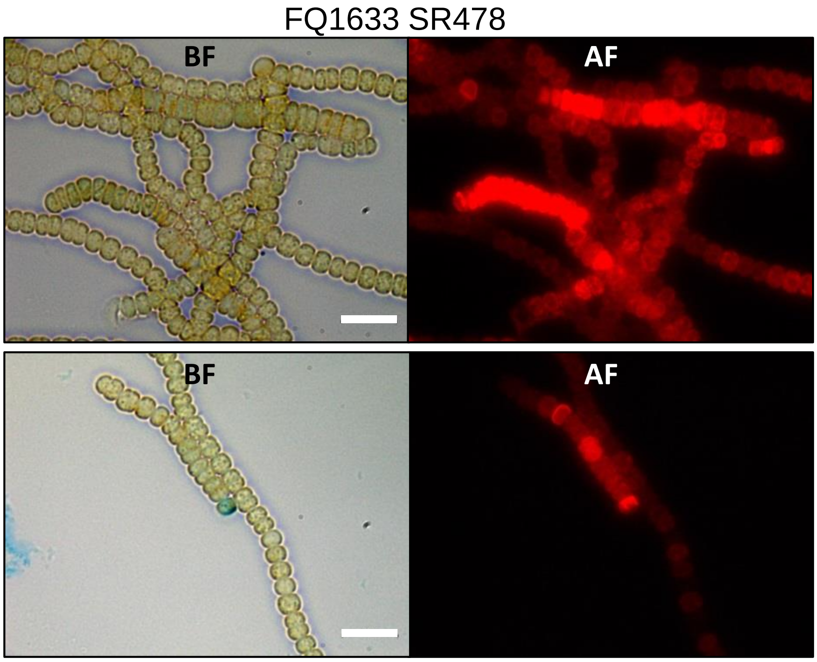

Figure S1. Morphology of the FQ1633 SR478 mutant strain. Alcian blue-stained filaments of the FQ1633 SR478 mutant 7 days after nitrogen deprivation. Brightfield images (BF) and images of auto-fluorescence (red; AF) are shown. Scale bars, 10 µm.

Figure S2

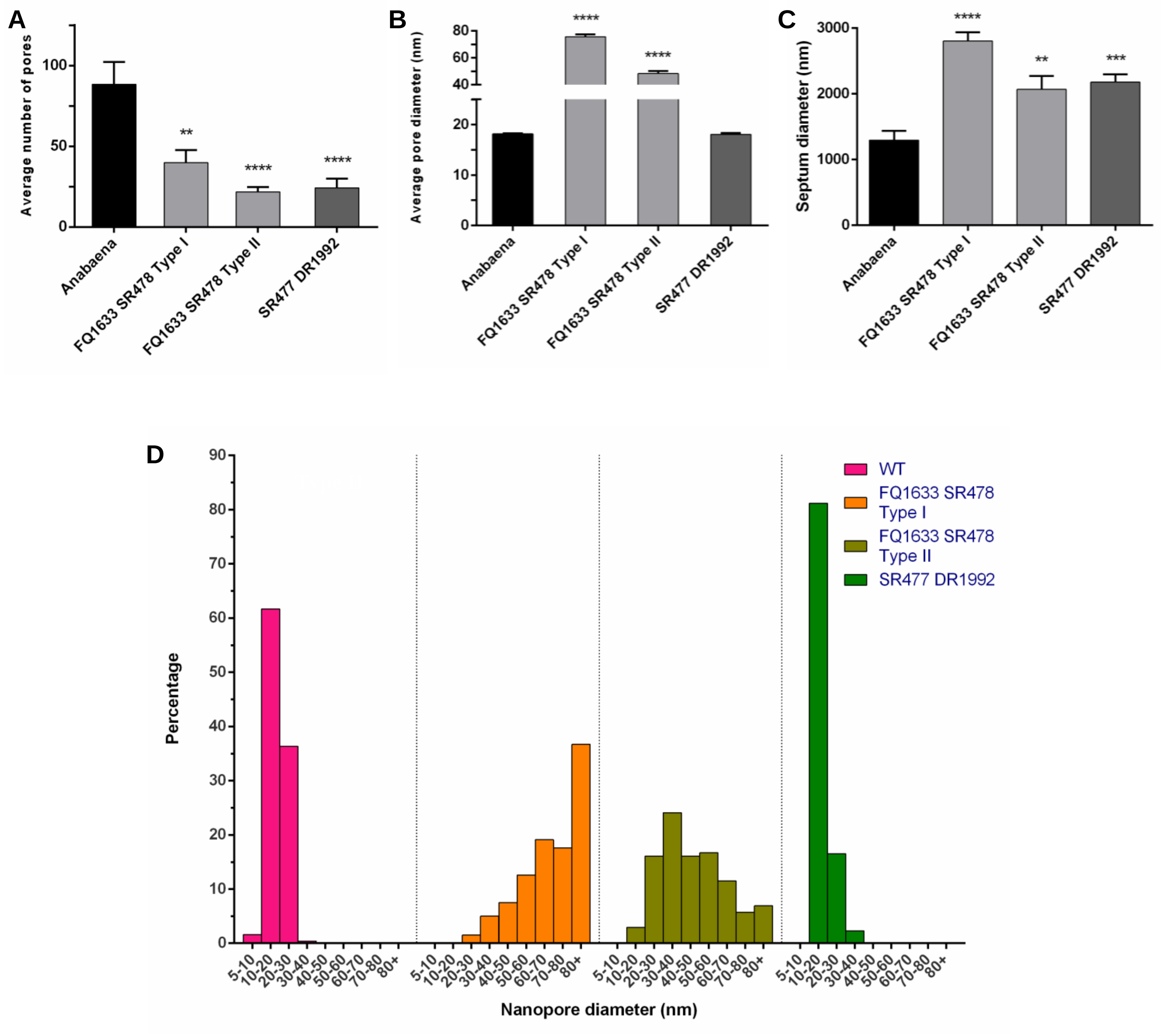

Figure S2. Septal nanopores in *Anabaena* and indicated *amiC1 amiC2* double mutants. (A) The histogram shows the mean number of nanopores per septum ± *SD* of the mean for the different strains and types of septa. Histogram (B) shows the average pore diameter ± *SD* of the mean for the indicated strains and types of septa. Histogram (C) shows the average septal diameter ± *SD* of the mean for the indicated strains and types of septa. Student's *t* test (mutant vs. wild type) *P* values are indicated as asterisks (** $P \leq 0.01$; *** $P \leq 0.001$; **** $P \leq 0.0001$). (D) Quantitative analysis of nanopore dimensions. Results were binned in 10-nm steps and the percentage of each bin is shown. Statistical values are given in Table S4.

Figure S3

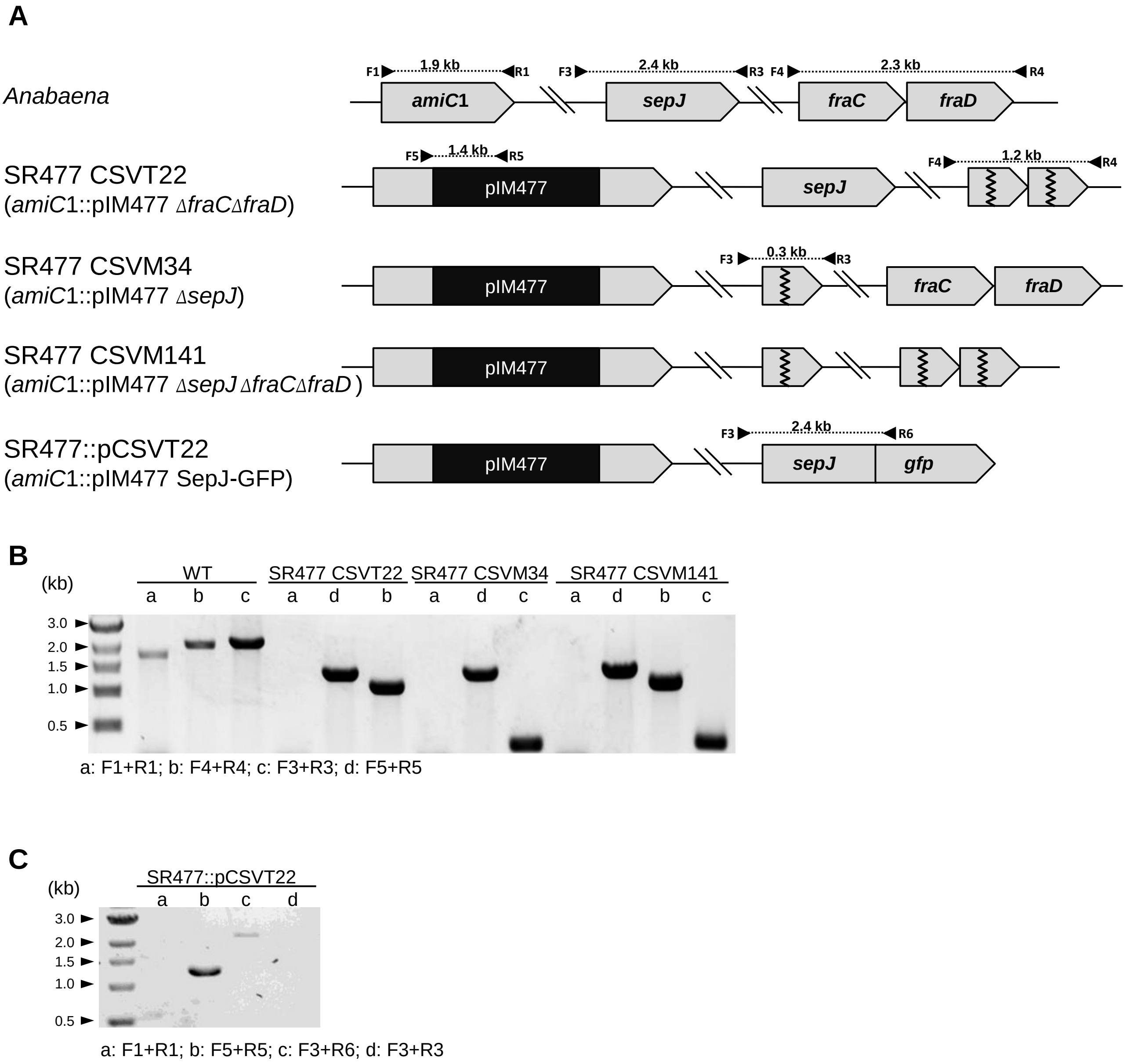

Figure S3. Structure of genomic regions of *Anabaena* and mutant strains used in this work. (A) Schematic of the genomic regions of *amiC1*, *sepJ* and *fraCD* in *Anabaena* and indicated mutant strains. PCR primers and expected product sizes are indicated. (B) Analysis of the *amiC1* inactivation in the indicated mutants. Primer-pair a, b and c illustrate if wild-type *amiC1*, *fraCD* and *sepJ* exist respectively. Primer-pair d illustrates integration of pIM477 into the *amiC1* locus. (C) Analysis of the SepJ-GFP fusion in the SR477 mutant background. Primer a and b illustrate if wild-type *amiC1* exists and integration of pIM477 into the *amiC1* locus respectively. Primer-pair c illustrates integration of *gfp* into the *sepJ* locus and primer pair d illustrates the knockout of wild-type *sepJ*. All primers are listed in table S2.
